# Seroepidemiological Investigation of Hepatitis B and C Prevalence and Associated Factors Among People in Custody at Zahedan Central Prison

**DOI:** 10.34172/aim.23553

**Published:** 2024-05-14

**Authors:** Maliheh Metanat, Seyedeh Zeinab Almasi, Nahid Sepehri Rad, Seyed Mehdi Tabatabaee, Kosar Rezaei

**Affiliations:** ^1^Infectious Diseases and Tropical Medicine Research Center, Research Institute of Cellular and Molecular Sciences in Infectious Diseases, Zahedan University of Medical Sciences, Zahedan, Iran; ^2^Health Promotion Research Center, Zahedan University of Medical Sciences, Zahedan, Iran; ^3^Department of Epidemiology and Biostatistics, School of Public Health, Tehran University of Medical Sciences, Tehran, Iran; ^4^Department of Epidemiology, Zahedan University of Medical Sciences, Zahedan, Iran

**Keywords:** Hepatitis B, Hepatitis C, Iran, People in custody, Prevalence, Risk factor

## Abstract

**Background:**

On a global scale, approximately 350 million are affected by hepatitis B, and 71 million by hepatitis C. People in custody face elevated risks for these infections. The prevalence and risk factors in Iranian prisons are insufficiently documented. The principal objective of this study was to ascertain the prevalence of hepatitis B and C, coupled with the identification of pertinent influencing factors, within the confines of Zahedan central prison, situated in the southeastern region of Iran.

**Methods:**

In 2019, we conducted an analytical cross-sectional study involving 407 people in custody, using stratified random sampling. To definitively diagnose hepatitis C virus (HCV) infection (*P*<0.05), a checklist developed by the researchers, along with enzyme-linked immunosorbent assay (ELISA) and polymerase chain reaction (PCR) techniques, were employed.

**Results:**

This study comprised 406 participants (96.3% male) with a median age of 32 years (27-38). Approximately 62% were married, and a substantial proportion of the participants had low education levels (47%), unemployment (64%), and belonged to the Baloch ethnicity (64%). The overall prevalence of hepatitis C and B infections was 2.7% and 10.6%, respectively. Tattooing (adjusted odds ratio [AOR]: 2.07, 95% CI: 1.9-4.5) and marriage (AOR: 1.78, 95% CI: 1.05-3.04) were identified as risk factors for hepatitis B. Moreover, hepatitis C showed a statistically significant association with a family history of hepatitis B and C (AOR: 3.31, 95% CI: 3.93-24.64) and intravenous (IV) drug use (AOR: 7.01, 95% CI: 1.52-32.78) according to the multivariable logistic regression analysis.

**Conclusion:**

The prevalence of hepatitis B and C was higher among people in custody in Zahedan central prison. Consequently, targeted interventions are vital to address and reduce viral hepatitis burden in custodial settings.

## Introduction

 Viral hepatitis poses a significant global public health challenge, contributing to approximately 1.4 million deaths annually.^1^ According to the World Health Organization (WHO) estimates in 2015, 257 million people worldwide were living with chronic hepatitis B, and 71 million had hepatitis C.^2^ Both hepatitis B virus (HBV) and hepatitis C virus (HCV) infections can progress to severe liver conditions, including cirrhosis and liver carcinoma.^3^ Transmission occurs primarily through infected blood and body fluid exposure such as semen, including intravenous (IV) drug use, vertical transmission, and unsafe healthcare practices.^4^ The substantial burden of viral hepatitis on healthcare systems underscores the urgency for effective interventions. In 2016, the WHO set a goal to eliminate hepatitis C as a public health concern by 2030.^1^

 Correctional facilities expose inmates to an elevated risk of viral infections, including viral hepatitis, due to prevalent high-risk behaviors like IV drug use, tattooing, and unprotected sexual relationships.^5^ HCV is particularly prevalent among people in custody, as a higher proportion engage in IV drug use compared to the general population.^6^ However, HCV prevalence in prisons varies widely in different studies, ranging from 1.1% to over 80%.^7,8^ In the European Union, anti-HCV prevalence among inmates ranges from 14.6% to 84.3%.^8^

 Iranian studies have also indicated varying prevalence rates of HCV and HBV among people in custody, underscoring the importance of further investigation in specific regions. For instance, studies in the Khorasan Razavi Province reported HCV and HBV seroprevalences of 24.5% and 4.2%, respectively, among people in custody compared to 19.1% and 2.1% in the general population.^9^ Additionally, investigations in other regions, such as Gorgan and Karaj, revealed HCV antibody and RNA prevalence rates ranging from 6.7% to 5.2% and 4.6% to 3.4%, respectively.^10,11^ A nationwide study covering 55 prisons in Iran found the prevalence of hepatitis B and C among people in custody with IV drug use to be 40.52% and 2.56%, respectively^12^ A meta-analysis published in 2016 estimated the prevalence of HBV infection in the general Iranian population to be 2.2%.^13^

 Considering the distinct characteristics and epidemiological variations between hepatitis B and C, it becomes imperative to prioritize the prevention and management of hepatitis C in Iran, particularly due to the absence of a vaccine.^2,14^ The implementation of the national hepatitis B vaccination program in Iran since 1993 has resulted in a change in transmission patterns, transitioning from vertical to horizontal transmission within the population.^15-17^ Despite existing studies in different regions, there is still lack of data concerning the seroprevalence of hepatitis B and C infections among people in custody in the Sistan and Baluchestan Province. Consequently, this study was conducted to investigate the prevalence of HBV and HCV infections among people in custody with a history of more than 6 months in Zahedan prison, with a focus on identifying associated risk factors.

## Materials and Methods

###  Study Population

 This analytical cross-sectional investigation was undertaken within Zahedan city in 2019. Ethical approval for this research endeavor was obtained from the Tropical Infectious Research Center at Zahedan University of Medical Sciences, bearing the ethics code (Project No. 9008-97). The study encompassed the entire incarcerated population within Zahedan city’s central prison, which consisted of a total of 2319 individuals in custody.

###  Data Collection 

 Upon obtaining approval for the research proposal, the investigators coordinated with the research department of Zahedan central prison to facilitate the implementation of the study. A specialized research team was assembled, consisting of two experts in public health, a nurse, and two laboratory science experts. To mitigate potential epidemiological errors, the team received comprehensive training in sample selection, data completion, and blood sampling procedures. Building a strong rapport with the study participants, the team emphasized the strict confidentiality of their information and clarified the research objectives. Written consent forms were duly obtained from all participants, ensuring their voluntary participation and understanding of the study’s purpose.

 Data collection involved employing a researcher-designed checklist, encompassing demographic details (age, sex, education level, ethnicity, marital status, occupation) and behavioral risk factors (polygamy, history of hepatitis B and C, family history of hepatitis B and/or C (Yes/No), history of blood transfusion (Yes/No) and coagulation factors (Yes/No), cupping therapy (Yes/No), tattooing (Yes/No), unprotected sexual contact (Yes/No), use of shared syringe/razor, drug use (Yes/No). The outcome variables comprised positive results for hepatitis B surface antigen (HBS-Ag) and anti-hepatitis B core antigen (anti-HBc) for hepatitis B, as well as positive HCV RNA (HCV-PCR) and anti-HCV for hepatitis C. Past infections were regarded as negative for HBV and HCV (anti-HCV positive & HCV-PCR negative, anti-HBc positive & anti-HBS positive). Data were gathered through face-to-face interviews.

###  Sample Size and Sampling

 After conducting a comprehensive literature review, the sample size was calculated to be 361 individuals based on the formula for cross-sectional studies (α: 0.05, β: 0.2, d: 0.05, p: 38%). To account for potential attrition, a total of 407 participants were selected using stratified sampling, with each ward being treated as a class. The prison had various sections, such as the kitchen section, education section, military section, and several numbered sections, along with health and women’s quarantine. Each of these sections was considered a class, and the number of samples required for each class was determined based on their respective sizes using stratified random sampling.

###  Laboratory Procedures

 Approximately 10 cc of venous blood was collected from each participant and sent for serum testing of HBsAg, anti-HBc, anti-HBS, and anti-HCV using ELISA kits (DiaPro, Italy). In case of positive antibody test results, HCV-RNA PCR was conducted for confirmation. Total RNA was extracted from the sera using Viral Nucleic Acid Extraction Kits (Roche Company, Germany) as per the manufacturer’s guidelines, followed by the use of CinnaGen diagnostic kits (CinnaGen Company, Iran) to continue the process. The PCR products were then analyzed on a 2% agarose gel containing ethidium bromide, and the results were assessed using gel documentation.

 In this study, we compared individuals diagnosed as positive cases of hepatitis during the research (with criteria for positive HBV being HBS-Ag and anti-HBc positive, and criteria for positive HCV being HCV-PCR positive and anti-HCV-positive) with others in the study.

###  Statistical Analysis

 Categorical variables were presented using counts and percentages. To compare the distribution of categorical variables among different groups, chi-square and Fisher’s exact tests were employed. The normality of continuous variables was assessed using Kolmogorov-Smirnov goodness-of-fit tests. Variables showing significant associations (*P* < 0.2) with hepatitis B and C infections in univariate logistic regression models were selected for inclusion in the final models. Multivariate logistic regression models were created using the forward likelihood ratio method to identify factors associated with hepatitis B and C infections. Statistical significance was defined as *P* < 0.05, and the analysis was conducted using STATA software version 16.

## Results

 Out of the initial 407 prisoners selected, 406 prisoners willingly participated in the study, yielding a response rate of 99%. The average age of the participants was 17.9 ± 18.33 years, and they had spent an average of 22.59 ± 42.01 months in prison. The majority of the participants were male (96.3%), and the most represented ethnicity was Baluch (65.8%). A significant proportion had limited education (49.6% were illiterate or had an elementary education), and a considerable portion were married (64.7%) and self-employed (67.3%). Moreover, the vast majority of participants had no history of polygamy (89.9%), no history of receiving blood, and only a small percentage had a history of receiving dental services (4.7%).

 A demographic analysis revealed that 33.4% of the participants fell within the age bracket of 18 to 28 years, while 65.5% of the participants indicated being married, and the majority had no family history of hepatitis B and C and had not engaged in shared needle use (95.5% for both). Additionally, 94.7% reported no history of injection addiction. As per the provided definitions, the overall prevalence of hepatitis B and hepatitis C was determined to be 10.6% (95% CI: 7-13) and 2.7% (95% CI: 0.8-15), respectively. The prevalence of past infection for HBV and HCV in the studied population was 26.8% and 6.1%, respectively. The results of HBV and HCV sero-epidemiological tests are presented in Table 1. Notably, all patients with HBV and HCV infections in our study were male.

**Table 1:** Seroepidemiological Interpretation in Prisoners

	**No. (%)**
Vaccinated (Anti-HBs)	202 (49.6)
Past infection/cleared (Anti hbc + & Anti HBS + )	109 (26.8)
HBV Infection (HBS Ag + )	43 (10.6)
Past Infection/cleared (Anti HCV + & HCV PCR-)	25 (6.1)
HcV Infection (Anti Hcv + & HCV PCR + )	11 (2.7)

###  Univariate Analysis

 Initially, we conducted univariate analyses to explore the association between various risk factors and hepatitis B and C infections. The results indicated significant associations between ethnicity (*P* = 0.02), age group (*P* = 0.01), education level (*P* = 0.02), marital status (*P* = 0.002), tattooing (*P* = 0.02), and cupping (*P* = 0.05) with hepatitis B. Additionally, family history of hepatitis B and C (*P* = 0.04), history of using shared needles (*P* = 0.02), tattoos (*P* = 0.01), injection addiction (*P* = 0.001), and unprotected sex (*P* = 0.05) were found to be associated with hepatitis C. For other variables, no compelling evidence was found to reject the hypothesis of their association with hepatitis B and C. The results tests are presented in Table 2.

**Table 2:** Risk Factors Associated with HBV and HCV Infection in Univariate Analysis Using Fisher’s and Chi-square Test

**Variables**		**HBV**^+^ **(N=43)**		**HCV**^+^ **(N=11)**	
	**Total**	**Median Interquartile**	***P*** ** Value**	**Median Interquartile**	***P*** ** Value**
Age	406	32 11	0.01	32 11	0.09
Length of imprisonment	406	12 32	0.87	12 32	0.15
		**No. (%)**		**No. (%)**	
Gender			0.17		0.65
Female	15	0 (0)		0 (0)	
Male	361	43 (11)		11 (2)	
Ethnicity			0.02		0.76
Baluch	261	16 (13)		8 (2)	
Persian	122	23 (8)		3 (3)	
Other	14	3 (21)		0	
Education			0.01		0.29
Illiterate and elementary	193	21 (10)		8 (4)	
Middle and high school	119	11 (9)		2 (1)	
Diploma and university	76	9 (11)		1 (1)	
Marital status			0.002		0.58
Single	142	12 (8)		4 (2)	
Married	252	31 (11)		7 (2)	
Job			0.05		0.62
Unemployed	41	3 (7)		1 (2)	
Manual worker	63	10 (15)		0 (0)	
Farmer	13	2 (15)		0 (0)	
Free job self-employed	263	27 (10)		10 (3)	
Employee	9	1 (11)		0 (0)	
Polygamy			0.35		0.73
Yes	41	3 (10)		2 (2)	
No	361	39 (7)		9 (2)	

 In the subsequent phase, after validating the assumptions of the multivariable logistic regression test, we assessed the relationship between the identified variables and hepatitis C, while controlling for potential confounders. To construct an appropriate multivariable model, six variables with *P* < 0.2 in the univariate tests (Chi-square test and Fisher’s test) were incorporated using the forward conditional method. These variables included age, family history of hepatitis B and C, history of shared needle and syringe use, tattooing, intravenous drug use, and unprotected sex. Similarly, for hepatitis B, 10 variables, namely age, sex, ethnicity, level of education, marital status, occupation, family history of hepatitis B and C, tattooing, and unprotected sex, were considered in the model (Table 3).

**Table 3:** Risk Factors Associated with HBV and HCV Infection in Univariate Analysis Using Fisher’s and Chi-square Test

**Variable**		**HBV (N=43)**		**HCV (N=11)**	
	**Total**	**No. (%)**	***P*** ** value**	**No. (%)**	***P*** ** value**
Family history of hepatitis B and C			0.12		0.04
No	385	38 (9)		9 (23)	
Yes	20	4 (21)		2 (10)	
Missing	1				
History of shared needle and syringe use			0.66		0.02
No	384	41 (10)		9 (2)	
Yes	19	2 (10)		2 (10)	
Missing	3				
Tattoo			0.02		0.01
No	189	13 (6)		1 (.05)	
Yes	218	30 (13)		10 (4)	
Cupping			0.05		0.52
No	343	32 (9)		9 (2)	
Yes	62	10 (16)		2 (3)	
Missing	2				
Injecting drug use			0.42		0.002
No	385	40 (10)		7 (1)	
Yes	22	3 (13)		4 (4)	
Unprotected sex			0.10		0.05
No	227	25 (9)		6 (1)	
Yes	130	18 (13)		5 (18)	

 The outcomes of the multivariable logistic regression revealed that individuals with a family history of hepatitis B and C were 3.31 times more likely to have hepatitis C (AOR: 3.31; 95% CI: 3.93-24.64) compared to the reference group, after adjusting for other variables. Moreover, IV drug use was associated with a 7.01-fold higher likelihood of hepatitis C (AOR: 7.01; 95% CI: 1.52-32.78) when adjusting for other variables. Regarding hepatitis B, tattooing and marriage were found to increase the odds of infection by 2.07 times (95% CI: 1.9-4.5) and 1.78 times (95% CI: 1.05-3.04), respectively, when adjusting for other variables (Table 4).

**Table 4:** Multivariable Logistic Regression Analysis of Factors Associated with the Prevalence of HCV and HBV Among Incarcerated People

**Variable**	**Adjusted OR (95% CL)**	***P*** ** value**
**HCV**		
Family history of hepatitis B and C		0.040
No	1 (Ref)	
Yes	3.31 (3.93-24.64)	
Injecting drug use		0.001
No	1 (Ref)	
Yes	7.01 (1.52-32.78)	
**HBV**		
Tattooing		0.03
No	1 (Ref)	
Yes	2.07 (1.9-4.5)	
Marital status		0.03
Single	1 (Ref)	
Married	1.78 (1.05-3.04)	

## Discussion

 This study represents a pioneering investigation into the prevalence of hepatitis B and C among people in custody (n = 407) in Zahedan. The findings indicate a higher prevalence of hepatitis B and C among the people in custody in Zahedan compared to the general population. The study identifies tattooing and marital status as risk factors for hepatitis B, while IV drug use is recognized as a risk factor for hepatitis C.

 The overall prevalence of hepatitis C and B in the study subjects was 2.7% and 10.6%, respectively, significantly higher than the rates observed in the general population. These findings align with previous research.^18,19^ Another study conducted in Iran reported a much higher prevalence of hepatitis C (18.6%) among people in custody,^20^ which could be attributed to differences in sample size and study population compared to our study. Furthermore, a study among IV drug users in Iran found a higher prevalence of hepatitis B (40.52%) and a comparable prevalence of hepatitis C (2.56%). The varying prevalence rates of hepatitis C in these studies may be attributed to the specific populations examined, considering significant risk factors such as IV drug use, prison history, and high-risk sexual behaviors.^18^

 In our study, no significant relationship between age and hepatitis C was observed, which is consistent with findings from a previous study.^21^ However, a study by Nokhodian et al in Isfahan prisoners reported a 4.4% prevalence of hepatitis C infection among juvenile prisoners.^22^ The lack of a significant relationship in our study may be attributed to its relatively low statistical power, despite having a large sample size. The number of individuals with the desired outcome (hepatitis C infection) was smaller than the number of individuals for each explanatory variable. On the other hand, our univariate analysis revealed a correlation between age and hepatitis B. The lower prevalence of hepatitis B among individuals aged 18-28 can be attributed to Iran’s national vaccination program implemented after 1993.^15^

 In our study, IV drug use emerged as an important risk factor for hepatitis C. The increasing prevalence of IV drug users in Iran has contributed to the changing epidemiology and rising incidence of hepatitis C.^23^ Similar findings have been reported in studies from other countries, highlighting IV drug use as a major risk factor for hepatitis C.^24,25^ This risk factor is common among people in custody worldwide. Studies have consistently shown that IV drug users have the highest prevalence of hepatitis C, which aligns with our study results.^26^ Lack of adequate harm reduction policies remains a significant challenge for people in custody. Implementing proper measures to identify IV drug users upon entry to prisons and providing them with sterile needles and syringes is essential to prevent further transmission of the infection within custodial settings.

 Our findings regarding tattooing as a risk factor for hepatitis B are consistent with previous research conducted in the Khorasan Razavi Province, Iran.^11^ A meta-analysis published in 2017 also identified tattoos as one of the primary risk factors for HCV transmission.^27^ Tattooing is a prevalent cosmetic technique worldwide and is commonly used for pain relief and beauty purposes in the Sistan and Baluchestan Province. However, if performed in a traditional and unsanitary manner, tattooing can facilitate the transmission of viral diseases through blood.^28^ In our univariate analysis, a significant association was observed between hepatitis C and tattooing. However, after adjusting for confounders in the final model, this relationship was no longer evident. On the other hand, in a cohort study conducted in Australia, a notable association was reported between tattooing and HCV infection.^29^ Our study similarly identified tattooing as a risk factor for hepatitis C infection, aligning with previous findings that suggest a higher prevalence of hepatitis C among tattooed individuals compared to the general population.^21^

 Our study did not find a significant association between gender and HCV infection, and a similar lack of significant relationship between males and females with hepatitis B was observed, consistent with findings from other studies.^30^ Notably, in the general Iranian population, a study reported a higher prevalence of HBV infection in males compared to females (3% vs. 1.7%).^9^ However, in our study, no difference in infection rates between genders was observed, as all individuals with hepatitis B and C were male, resulting in a zero prevalence of the disease in females.

 Our study revealed a considerably higher prevalence of hepatitis B among people in custody (10.6%) compared to the seroprevalence of 2.5% in the general population of Zahedan.^19^ This finding highlights that prisons can act as environments conducive to the transmission of infections, necessitating targeted efforts to combat hepatitis C and hepatitis B among people in custody. Additionally, the overall prevalence of HBV infection among high-risk groups in Iran is approximately 4.8%, which is double the rate observed in the general Iranian population (i.e. 2.2%).^9^

 In our study, the likelihood of contracting hepatitis B was 1.78 times higher among married individuals compared to those who were single. This observation aligns with the findings of another study^31^ that highlighted marriage and heterosexual relationships as significant risk factors for hepatitis transmission. Furthermore, our multivariate analysis revealed a significant association between hepatitis B and unprotected sexual activity, consistent with a prior study conducted in Birjand, Iran.^32^ Our results provide support for the potential transmission of hepatitis B through sexual contact. Given the incarceration context, where individuals are separated from their families and considered high-risk groups, there is a heightened concern regarding their susceptibility to infections and the potential for transmitting the virus to their spouses through sexual relationships.

 The formulation of HCV control policies involves numerous stakeholders, each with varying levels of engagement and support in the decision-making process. It is essential for these policies not to be solely dictated by the Ministry of Health and Medical Education, but rather to be endorsed and supported by all relevant and competent stakeholders.^33^ The notable decline in HBV prevalence among IV drug users is attributed to the implementation of public health interventions targeted at both HIV and hepatitis B. Commencing in 2002 and expanded in 2005, harm reduction measures encompassed needle and syringe programs alongside opioid replacement therapy.^34^

 Our study did not reveal significant associations between certain well-known risk factors for hepatitis B and C and the prevalence of infection in our study population. However, it did highlight a correlation between low levels of education and higher prevalence of HBsAg, consistent with findings from a prior study that identified the absence of a high-school diploma as a predictor of HBV infection.^27^ Nevertheless, the contrasting results from another study conducted in Zahedan^19^ may be attributed to variations in the studied groups. Thus, people in custody, who are more likely to have lower education levels, appear to face an elevated risk of infection compared to the general population. In our study and another previous investigation in Zahedan,^19^ self-employed individuals and those of Baluch ethnicity demonstrated a higher hepatitis prevalence, yet the impact of ethnicity could not be confirmed in multivariable analysis.

## Conclusion

 In conclusion, our study highlights a concerning prevalence of hepatitis B and C among people in custody, emphasizing the importance of preventive programs. Although the overall prevalence of hepatitis C in Iran remains low, the epidemiology is evolving with a rise in incidence due to an increase in injecting drug users in the community. Our findings identify tattooing and marital status as risk factors for hepatitis B, while IV drug use is a significant risk factor for hepatitis C. To combat this issue, we recommend implementing preventive measures to reduce high-risk behaviors, establishing effective communication with patients for early treatment, promoting immunization, safe tattooing practices, and providing addiction treatment services. These efforts are crucial in controlling the spread of viral hepatitis in custodial settings.
